# A two-stage amplified PZT sensor for monitoring lung and heart sounds in discharged pneumonia patients

**DOI:** 10.1038/s41378-021-00274-x

**Published:** 2021-07-22

**Authors:** Hongbin Chen, Shuai Yu, Haiyang Liu, Jie Liu, Yongguang Xiao, Dandan Wu, Xiaoyu Pan, Cuihong Zhou, Yifeng Lei, Sheng Liu

**Affiliations:** 1 Department of Pulmonary and Critical Care Medicine, Renmin Hospital of Wuhan University, Wuhan, 430060 China; 2 School of Mechanical Science and Engineering, Huazhong University of Science and Technology, Wuhan, 430074 China; 3 School of Power and Mechanical Engineering & the Institute of Technological Science, Wuhan University, Wuhan, 430072 China; 4 Department of Thoracic, Renmin Hospital of Wuhan University, Wuhan, 430060 China; 5 Department of Pulmonary and Critical Care Medicine, the Ninth Hospital of Wuhan, Wuhan, 430081 China; 6 School of Microelectronics, Wuhan University, Wuhan, 430072 China

**Keywords:** Electrical and electronic engineering, Materials science

## Abstract

Assessment of lung and heart states is of critical importance for patients with pneumonia. In this study, we present a small-sized and ultrasensitive accelerometer for continuous monitoring of lung and heart sounds to evaluate the lung and heart states of patients. Based on two-stage amplification, which consists of an asymmetric gapped cantilever and a charge amplifier, our accelerometer exhibited an extremely high ratio of sensitivity to noise compared with conventional structures. Our sensor achieves a high sensitivity of 9.2 V/g at frequencies less than 1000 Hz, making it suitable to use to monitor weak physiological signals, including heart and lung sounds. For the first time, lung injury, heart injury, and both lung and heart injuries in discharged pneumonia patients were revealed by our sensor device. Our sound sensor also successfully tracked the recovery course of the discharged pneumonia patients. Over time, the lung and heart states of the patients gradually improved after discharge. Our observations were in good agreement with clinical reports. Compared with conventional medical instruments, our sensor device provides rapid and highly sensitive detection of lung and heart sounds, which greatly helps in the evaluation of lung and heart states of pneumonia patients. This sensor provides a cost-effective alternative approach to the diagnosis and prognosis of pneumonia and has the potential for clinical and home-use health monitoring.

## Introduction

Assessment of lung and heart states is critical when evaluating the health condition of patients with pneumonia. Lung injury in patients can be revealed by abnormal findings based on chest CT images^1–3^, PET/CT^4^, and artificial intelligence (AI)-assisted diagnosis^5,6^. Lung ultrasound also offers a quantitative method to assess the lung state in patients^7^. Meanwhile, heart injury in patients can be revealed by echocardiography (ECG)^7^ and cardiac magnetic resonance imaging (MRI)^8,9^. However, these methods generally require large, sophisticated, and expensive instruments; highly trained personnel; complex procedures; and far less harmless procedures (such as CT and MRI). Therefore, the development of novel sensing systems that are time-saving, low cost, highly sensitive, easy to read, instrument-free, and able to achieve on-site continuous monitoring^10,11^ has great potential in the diagnosis and prognosis of pneumonia diseases.

Auscultation of chest wall sounds, including both heart and lung sounds, offers an easy but very effective approach for the clinical diagnosis of cardiovascular and respiratory systems. Conventional stethoscopy is widely used for intermittent auscultation; however, stethoscopy has a number of limitations, such as poor wearability due to its bulky size, friction noise during diagnosis, and difficulty in detecting weak acoustic signals including lung sounds. An alternative approach for detecting lung and heart sounds is based on accelerometer use^12,13^. Compared with stethoscopy, miniaturized accelerometers can be taped on a person’s chest wall for more convenient and continuous cardiorespiratory monitoring. Previously, based on asymmetric gapped cantilever structures, we developed a series of small-sized and ultrasensitive sound sensors for continuous monitoring of heart and lung sounds in healthy subjects^14–16^. However, none of them have been systematically used to monitor patients with pneumonia.

Herein, we were motivated to explore more applications of our sound sensors in the assessment of lung and heart states of discharged pneumonia patients. From both theoretical simulations and mechanical tests, our sensors show improved sensitivity compared with conventional sensors, making them suitable for monitoring weak heart and lung sounds. Moreover, the lung and heart sound recorded by our sensors are in good agreement with previous clinical reports, suggesting that our sensor offers a potential alternative for the diagnosis and prognosis of pneumonia or other similar diseases.

## Results and discussion

### Sensor structure and working principles

**Fig. 1:**
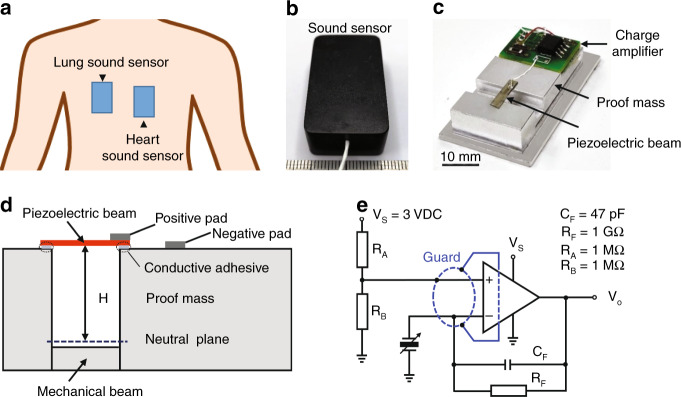
Cardiorespiratory sound sensors and sensing mechanism. **a** Illustration of the placement of sensors on the human body for lung and heart sound monitoring. **b** Image of the prototype of the sound sensor. **c** Inside view of the prototype with a printed circuit board. **d** Structure of the accelerometer-based on an asymmetric gapped cantilever structure. *H* is the distance between the layer of the piezoelectric beam and the neutral plane. **e** Built-in charge amplifier circuit for amplification of the piezoelectric signal

In this study, we used a self-developed sound sensor with high sensitivity for continuous monitoring of lung and heart sounds (Fig. 1a, b). The sound sensor was based on a novel asymmetric gapped cantilever structure (Fig. 1c, d), which was composed with a piezoelectric beam made of piezoelectric ceramic materials of lead zirconium titanate (PZT) as the top layer, a bottom mechanical layer separated by a gap, and a movable proof mass made of aluminum (Table 1). The piezoelectric layer could convert biomechanical energy (such as acoustic vibration) to electric energy due to the piezoelectric effect^17,18^. The mechanical beam could strengthen the stiffness of the whole cantilever (Fig. 1d). Furthermore, the output of the sound signal was amplified using an amplifier circuit (Fig. 1e).

**Table 1:** Parameters of materials used for our sensor

	Materials	Density (kg/m^3^)	Young’s modulus (GPa)	Size (mm)
Piezoelectric beam	PZT	7.8 × 10^3^	66	3 × 1 × 0.127
Mechanical beam	Aluminum	2.7 × 10^3^	69	3 × 12 × 0.38
Proof mass	Aluminum	2.7 × 10^3^	69	20 × 12 × 1.5

### Theoretical simulation and characterization of sensor performance

**Fig. 2:**
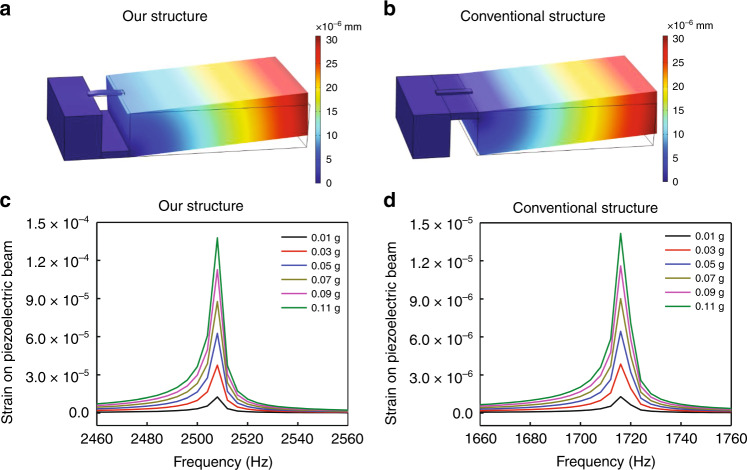
Theoretical simulation of sensor performance with different structures. **a**, **b** Total displacement of the sensor with our structure (**a**) or with a conventional structure (**b**); the excitation is 0.05 g. **c**, **d** Amplitude-frequency response of the sensor with our structure (**c**) or with a conventional structure (**d**); different excitation forces are simulated

We used theoretical simulation to show the advantage of our sensor with an asymmetric gapped cantilever structure compared with conventional structures (Fig. S1). Harmonic response analysis of the dynamic model was conducted under different excitation accelerations (from 0.01 g to 0.11 g). According to theoretical simulation, the strain experienced by the piezoelectric beam on our structure (Fig. 2a) was much more significant than that on a conventional structure (Fig. 2b). In our asymmetric gapped cantilever structure, the amplitude-frequency response showed that the maximum strain on the piezoelectric beam was 1.38 × 10^−4^ under 0.11 g excitation (Fig. 2c). In contrast, the maximum strain on the piezoelectric beam of the conventional structure was only 1.42 × 10^−5^ under the same excitation (Fig. 2d). Therefore, according to theoretical simulation, our structure produced a ten times higher strain on the piezoelectric beams than the conventional structure (Fig. 2).

**Fig. 3:**
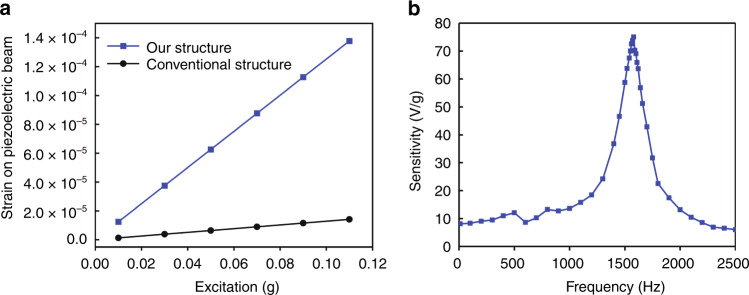
Sensitivity of the sensors. **a** Sensitivity of sensors with different structures by theoretical simulation. **b** Sensitivity of our sensor measured on a mechanical shaker

We then plotted the strain-excitation response of different structures (Fig. 3a). The sensitivity of the accelerometer can be defined by the equation below:

$$sensitivity = \frac{{strain\,of\,piezoelectric\,beam}}{{excitation\,force}}$$

Therefore, the sensitivity of the accelerometer can be calculated from the slope of the strain-excitation response (Fig. 3a), and the sensitivity of the accelerometer with our structure and conventional structure was calculated to be 1.25 × 10^−3^ and 1.29 × 10^−4^ (1/g), respectively. The theoretical simulation indicates that the sensitivity of our sensor structure was improved 9.7 times that of the accelerometer with a conventional structure.

The strain experienced by the piezoelectric layer is proportional to the distance (*H*) between the top piezoelectric beam and the neutral plane (Fig. S1)^19^. This distance is much larger on our structure than that on a conventional cantilever-based accelerometer (Fig. S1). This explained the significantly larger strain or higher sensitivity of our sensor compared with that of conventional accelerometers.

Moreover, due to the piezoelectric effect^17,18^ of PZT materials, the strain on the piezoelectric beam can be transferred into an electric charge. As the charge produced by the piezoelectric beam was very weak and could not be directly collected, we used a charge amplifier to transfer the charge into voltage and further amplify the signal (Fig. 1c, e); therefore, the sensitivity of the accelerometer can be expressed as V/g.

To avoid using balanced dual supplies to create op-amp circuits, the op-amp LMP7721 (Texas Instrument), which enables a single supply, was selected for the charge amplifier design (Fig. 1e). LMP7721 has an ultralow typical input bias current of 3 fA and low voltage noise of 6.5 nV/√Hz, making it ideal for amplifying high impedance signals. The average level of op-amp input was biased to *V*_*S*_/2 by the *R*_*A*_–*R*_*B*_ divider pair (Fig. 1e). The amplification rate of this circuit is inversely proportional to the feedback capacitance (*C*_*F*_). The signal-to-noise ratio (SNR) and lower cutoff frequency are also inversely proportional to the *C*_*F*_. The *C*_*F*_ in this charge amplifier was set as 47 pF (Fig. 1e) at the tradeoff of charge amplification rate, SNR, and lower cutoff frequency. Since the input capacitance of the piezoelectric transducer is ~1 nF, the amplification rate of the circuit was calculated to be 21.3. In addition, the charge amplifier circuit was designed with a 1 GΩ feedback resistor (*R*_*F*_) (Fig. 1e). Together, this circuit yielded a low cutoff frequency of 3.4 Hz, making it satisfactory for heart and lung sound monitoring.

In addition, ultralow input bias current op-amp circuits require precautions to achieve the best performance. The leakage current on the surface of the circuit board could exceed the input bias current of the amplifier and could even be 100 times higher. To minimize surface leakage, a guard trace was designed to completely surround the input terminals and other circuitry connecting to the inputs of the op-amp (Fig. 1e).

### Measurement of the sensitivity and noise of our sensor

We measured the sensitivity of our sensor on a mechanical shaker by setting a commercial accelerometer (752A13, Endevco) as the gold standard. The sensitivity-frequency response of our sensors is shown in Fig. 3b. Our sound sensor had a resonance frequency of 1600 Hz (Fig. 3b), which is higher than the frequency range of heart sounds (20–400 Hz) and lung sounds (60–1000 Hz).

**Fig. 4:**
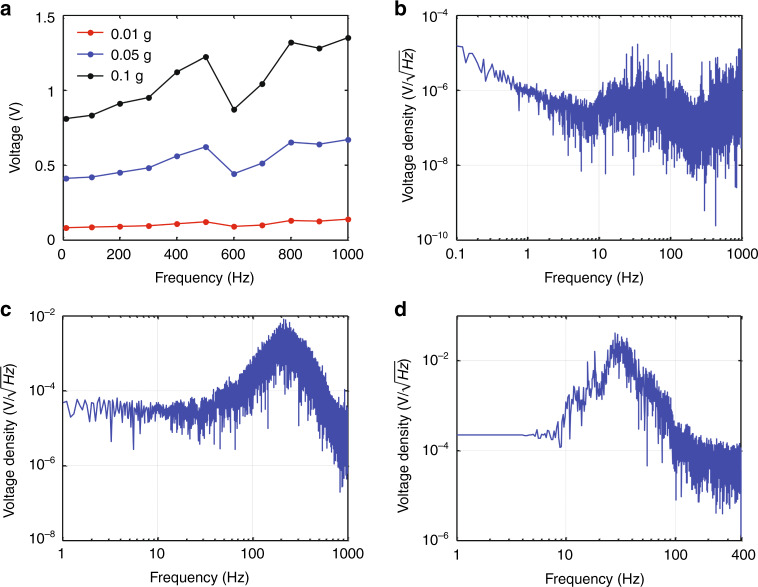
Sensor performance at sound frequencies ranging from 20 to 1000 Hz. **a** Output voltage response to different excitation accelerations. **b** Noise spectrum measured upon a vibration isolation mechanical shaker. **c** Lung sound signal spectrum and (**d**) heart sound signal spectrum measured on a healthy subject

As shown in Fig. 4a, within the sound frequency range from 20 to 1000 Hz, the output voltage of our sensor increased when the excitation acceleration increased. The sensitivity of our sensor at 0.01 g, 0.05 g, and 0.1 g under different excitation accelerations was calculated to be 9.1966 V/g, 9.1982 V/g, and 9.252 V/g, respectively (Fig. 4a). These results proved that the sensitivity of our sensor was consistent (~9.2 V/g) under different excitation accelerations within the heart and lung sound frequency ranges.

By using the novel asymmetric gapped cantilever structure, the sensitivity of our sound sensor could achieve 9.2 V/g at frequencies less than 1000 Hz (Figs. 3b and 4a). The sensitivity of conventional piezoelectric accelerometers or MEMS-based accelerometers is generally less than 1 V/g (Table 2)^20–22^. Comparably, our sensor showed significantly improved sensitivity (9.2 V/g) in frequencies less than 1000 Hz. The enhanced sensitivity of our sensor makes it suitable for the detection of weak physiological sounds, such as lung and heart sounds, especially for weak lung sound detection^23^.

**Table 2:** Comparison of different types of sound sensors

Sensor	Mechanism	Size	Weight	Sensitivity
Our sensor	Piezoelectric accelerometer	*l*: 39 mm *w*: 23 mm *h*: 13.5 mm	13.5 g	9200 mV/g
Endevco 752A12	Piezoelectric accelerometer	*h*: 23.6 mm	13 g	100 mV/g
PCB 393 C	Piezoelectric accelerometer	*Φ*: 57.2 mm *h*: 54.9 mm	885 g	1000 mV/g
Analog devices ADXL354	MEMS accelerometer	*l*: 6 mm *w*: 6 mm *h*: 2.1 mm	0.26 g	400 mv/g
Bosch Sensortec BMA456	MEMS accelerometer	*l*: 2 mm *w*: 2 mm *h*: 0.65 mm	<0.1 g	300 mV/g

In addition, since sensor noise is another important characteristic, we measured the intrinsic noise of our sensor upon a vibration isolation mechanical shaker at midnight. As shown in Fig. 4b, the noise spectrum and the output voltage density demonstrated that the noise level of our sensor was ~1 μV/√Hz within the frequency range of heart and lung sounds (from 20 to 1000 Hz). Therefore, the lower noise limit of our sensor was calculated to be 109 ng/√Hz.

From the lung sound signal spectrum (Fig. 4c), the output voltage density of the lung sound signal from 60 to 1000 Hz was ~125 μV/√Hz (Fig. 4c), which was 125 times higher than the intrinsic noise level. Similarly, from the heart sound signal spectrum (Fig. 4d), the output voltage density of the heart sound signal was ~890 μV/√Hz from 20 to 400 Hz (Fig. 4d), which was 890 times higher than the intrinsic noise level.

The SNR can be calculated according to the equation below,

$${\mathrm{SNR}} = 20\,{\mathrm{log}}_{10}\left( {Vs/Vn} \right)$$

where *Vs* and *Vn* represent the signal voltage and noise voltage, respectively. Therefore, the SNRs of the lung sound signal and heart sound signal was 42 dB and 59 dB, respectively. The SNRs of our sensors are two times higher than those of commercial stethoscopes.

### Sensor performance for lung and heart sound monitoring

Subsequently, we used our sound sensor device to monitor the lung and heart sounds of healthy volunteers in a regular laboratory environment to prove the auscultation ability of our sensor device.

**Fig. 5:**
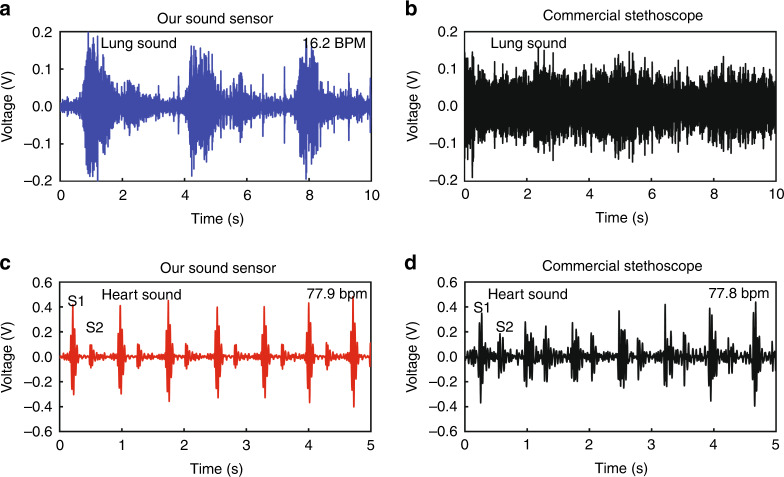
Waveforms of lung and heart sounds monitored on a healthy volunteer. **a**, **b** Lung sound waveform detected by our sound sensor (**a**) and by a commercial high-end electric stethoscope (**b**). **c**, **d** Heart sound signal recorded by our sensor (**c**) and by commercial stethoscopy (**d**). The volunteer was a 29-year-old healthy male (#1), and sound signals were recorded during a resting respiratory function

Compared with a commercial high-end electric stethoscope based on a conventional cantilever structure, our sensor exhibited much better performance for recording both lung and heart signals, and especially for recording weak lung sounds (Fig. 5). Generally, lung sounds are much weaker than heart sounds during regular breathing^23^; therefore, lung sounds, especially for gentle breathing, are difficult to detect. However, with the asymmetric gapped cantilever structure, our sensor can indeed detect weak lung sounds with a high SNR (Fig. 5a, Audio S1). In contrast, the commercialized high-end electronic stethoscope, which is based on a conventional cantilever structure, can hardly distinguish lung sounds from the noise of the captured signal (Fig. 5b). The respiratory rate of the measured volunteer was 16.2 breaths per minute (hereafter “BPM”) (Fig. 5a, Table 3), which was in the normal range of resting respiratory rates (12–20 BPM). Moreover, the lung sounds measured from different healthy volunteers were consistently within the normal respiratory rate range (Table 3).

**Table 3:** Monitoring of lung and heart sounds in healthy volunteers by our sound sensor

Healthy volunteers	Respiratory rate by sensor (BPM)	Heart rate by sensor (bpm)
#1	16.2	77.9
#2	17.1	72.3
#3	15.8	69.0
#4	18.2	75.0
#5	15.4	70.6

For heart sound monitoring, the SNR of the heart sounds detected by our sensor was two times higher than that of a commercial stethoscope (Fig. 5c, d). We could clearly distinguish two normal heart sounds from the obtained heart sound waveform, including the first heart sound (S1) and the second heart sound (S2) (Fig. 5c, Audio S2), which correspond to the “lub” and “dub” sounds of a heartbeat, produced by the closure of the atrioventricular valves and semilunar valves, respectively^24^. The heart rate of the measured healthy volunteer was 77.9 beats per minute (hereafter “bpm”) (Fig. 5c and Table 3), which was in the normal heart rate range of adults (60–88 bpm). Moreover, the heart sounds from different healthy volunteers were consistently within the normal range of heart rates (Table 3).

The measurements of healthy volunteers proved that our sound sensor could effectively detect lung and heart sounds in the human body (Table 3), especially relatively weak lung sounds (Fig. 5a). It is also worth noting that these measurements were carried out in a regular laboratory environment full of airborne noise. These results proved that our sensor was not very sensitive to airborne noise and can therefore be applied in medical applications.

### Sensor monitoring of patients with pneumonia

#### Classification of lung and heart sounds of patients

We monitored the lung and heart sounds of discharged pneumonia patients during their follow-up visit to the hospital to evaluate their lung and heart states.

According to the sensor monitoring and based on the clinical diagnosis of the discharged pneumonia patients, we found four typical characteristics from the recorded sound signals (Table 4) independent of the patients’ sex, age, preexisting conditions, the severity of illness, and time from the original diagnosis. The four types were typing I, patients with normal respiratory rate and normal heart rate; type II, patients with shortness of respiratory but normal heart rate; type III, patients with the normal respiratory rate but high heart rate; and type IV, patients with shortness of respiratory and high heart rate (Table 4).

**Table 4:** Definition of four types of lung and heart states in patients

	Respiratory rate (BPM)	Heart rate (bpm)
Type I	12–20	60–88
Type II	>20	60–88
Type III	12–20	>88
Type IV	>20	>88

Generally, a decreased respiratory rate is a good sign for healthy adults^23^, and the heart rates of some athletes or those who often exercise may be lower than those of ordinary adults^23^, which may explain why a decreased respiratory rate or decreased heart rate were hardly found in discharged pneumonia patients in this study.

#### Characterization of lung and heart sounds of patients

We described our findings of these four types of lung and heart sounds in detail.

**Fig. 6:**
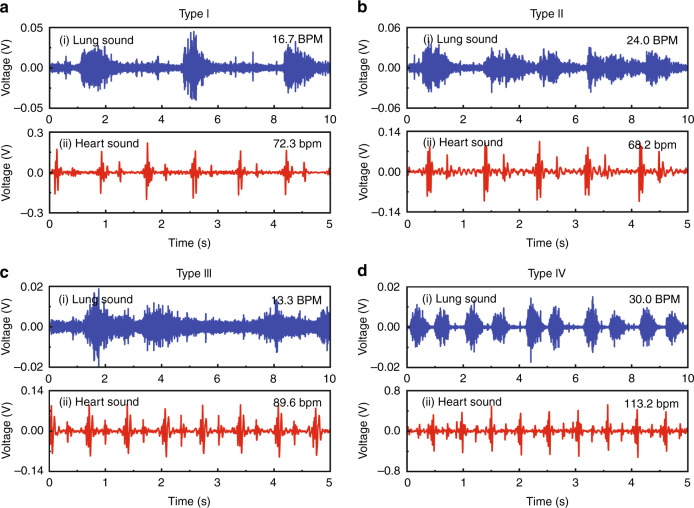
Four types of lung and heart sound recorded from discharged pneumonia patients. **a** Records of a patient with a normal respiratory rate and normal heart rate (#31). **b** Records of a patient with shortness of breath but a normal heart rate (#3). **c** Records of a patient with a normal respiratory rate but a high heart rate (#16). **d** Records of a patient with shortness of breath and a high heart rate (#4). **a**_**i**_, **b**_**i**_, **c**_**i**_, **d**_**i**_ Lung sounds detected by our sensor; **a**_**ii**_, **b**_**ii**_, **c**_**ii**_, **d**_**ii**_ Heart sounds recorded by our sensor

Type I patients exhibited both a normal respiratory rate and normal heart rate (Fig. 6a, Audio S3–S4). From our sensor monitoring, both the respiratory rate and heart rate of the patient were in normal ranges (Fig. 6a_i_, a_ii_, Table S1). We found that the lung and heart sounds of most discharged patients (29/41) exhibited type I characteristics, with an average respiratory rate of 16.5 ± 1.8 BPM and an average heart rate of 70.9 ± 6.5 bpm (Table 5), where the latter showed no significant difference from the ECG data (*p* > 0.05, Table 5). This result indicated that most discharged patients (70.7%) recovered from pneumonia and exhibited good lung and heart functions after discharge (Table 5).

**Table 5:** Four types of lung and heart states recorded in discharged pneumonia patients

	Respiratory rate by sensor (BPM)	Heart rate by sensor (bpm)	Number of patients	Ratio of patients	Heart rate by ECG (bpm)	*p* value among heart rates
Type I	16.5 ± 1.8	70.9 ± 6.5	29	70.7%	71.7 ± 6.4	0.0718
Type II	22.9 ± 1.5	75.9 ± 6.4	6	14.6%	76.2 ± 5.6	0.0504
Type III	12.9 ± 0.6	88.9 ± 0.9	2	4.9%	89.5 ± 3.5	0.0525
Type IV	30.0 ± 1.2	98.8 ± 10.7	4	9.8%	97.8 ± 8.1	0.0505

Type II patients showed shortness of respiratory function but normal heart rates (Fig. 6b, Audio S5–S6). From our sensor monitoring, the respiratory rate of the monitored patient increased to 24.0 BPM (Fig. 6b_i_), and the heart rate of the patient was 68.2 bpm (Fig. 6b_ii_, Table S1). We found 6/41 discharged patients with type II characteristics (Table 5), with an average respiratory rate of 22.9 ± 1.5 BPM and an averaged heart rate of 75.9 ± 6.4 bpm (Table 5), respectively. The measured heart rates were in good accordance with the ECG data (*p* > 0.05, Table 5). This result showed that 14.6% of the discharged patients exhibited normal heart function but still suffered from impaired lung function due to pneumonia infection (Table 5).

Type III patients exhibited a normal respiratory rate but an increased heart rate (Fig. 6c, Audio S7–S8). Our sensor monitoring showed that these patients exhibited a normal respiratory rate (13.3 BPM) (Fig. 6c_i_) but with an increased heart rate (89.6 bpm) (Fig. 6c_ii_, Table S1). From our sensor monitoring, we found 2/41 patients with type III characteristics (Table 5), with an average respiratory rate and heart rate of 12.9 ± 0.6 BPM and 88.9 ± 0.9 bpm, respectively (Table 5). These observations indicate that 4.9% of the discharged patients exhibited recovered lung function but still faced a critical challenge of heart injury (Table 5).

Type IV patients showed the worst recovery and exhibited both shortness of respiratory function and increased heart rate (Fig. 6d, Audio S9–S10). Our sensor monitoring showed that a patient’s respiratory rate increased to 30.0 BPM (Fig. 6d_i_) and the patient’s heart rate increased to as high as 113.2 bpm (Fig. 6d_ii_ and Table S1). We found 4/41 patients with type IV characteristics (Table 5), with an average respiratory rate of 30.0 ± 1.2 BPM, and an averaged heart rate of 98.8 ± 10.7 bpm (Table 5). These results indicated that 9.8% of discharged patients had very poor recovery and suffered from both heart injury and lung injury after pneumonia infection (Table 5).

Generally, lung injury during pneumonia infection is revealed by chest CT imaging^2,3^ and lung ultrasound^7^. Even after patients with pneumonia are discharged, patients may suffer from a lung injuries, such as lung fibrosis and changes in lung function^1^. Compared with sophisticated CT or lung ultrasound instruments, our small-sized sound sensor provided a fast and effective evaluation of lung function of the patients and revealed that 24.4% of the discharged pneumonia patients had lung injury in terms of shortness of respiratory function (type II and IV, Table 5).

In addition, pneumonia prominently affects the cardiovascular system of patients^8^. The presence of cardiac injury and myocardial inflammation in patients recovered from pneumonia was revealed by ECG^7^ and cardiovascular MRI^9^. From the heart sounds measured by our sound sensor, we also revealed heart injury in 14.7% of the discharged patients (type III and IV, Table 5). Compared with conventional ECG and cardiac MRI, our sensor provides a simple, easy but very effective approach to evaluate heart injury in discharged patients.

#### Time course tracking of the lung and heart state of a pneumonia patient

We next tracked the lung and heart sounds of a patient at different time points after discharge to evaluate the time evolution of the lung and heart states of the patient.

**Fig. 7:**
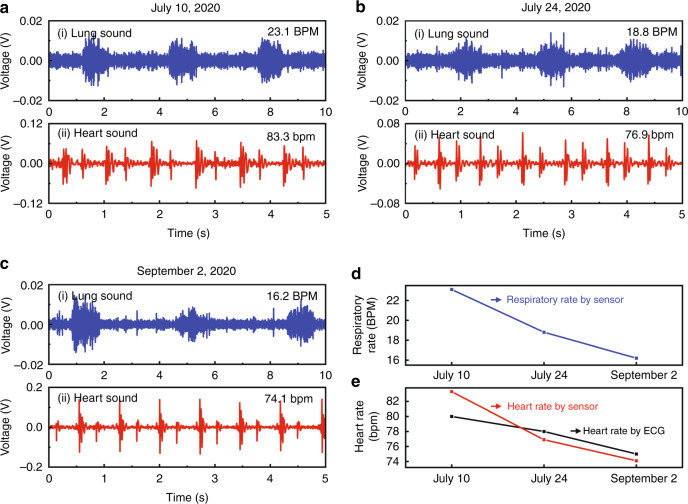
Time evolution of lung and heart states of a discharged pneumonia patient. **a**, **b**, **c** Sound sensor monitoring of the patient (#23) on July 10 (**a**), July 24 (**b**), and September 02, 2020 (**c**). **a**_**i**_, **b**_**i**_, **c**_**i**_ Lung sounds recorded by our sensor; **a**_**ii**_, **b**_**ii**_, **c**_**ii**_ Heart sounds detected by our sensor. **d** Time evolution of lung sounds of the patient detected by sensor monitoring. **e** Time evolution of heart sounds of the patient detected by our sound sensor monitoring (red curve) compared with ECG data (black curve)

Over time, from our sound sensor monitoring, we found that the lung and heart function of the monitored patient gradually improved (Fig. 7). From the lung sound waveforms, the respiratory rate of the monitored patient decreased from 23.1 to 18.8 and then to 16.2 BPM on different dates (Fig. 7a_i_, b_i_, c_i_, d), changing from shortness of breath to a normal respiratory rate. These observations suggested that the lung function of the patient gradually improved to a normal state. Moreover, from the monitoring of heart sounds, the heart rate of the patient was 83.3, 76.9, and 74.1 bpm on different dates, respectively (Fig. 7a_ii_, b_ii_, c_ii_, e and Table S2), indicating the improvement of the heart states of the patient.

#### Time evaluation of lung and heart states of 41 pneumonia patients

We then investigated the time evolution of lung and heart states of discharged pneumonia patients (*n* = 41). During the first monitoring session on 15 June 2020, the ratio of four types of patients was evenly distributed (Tables S3–S4 and Fig. S2a). Over time, the ratio of type I patients increased, whereas the ratio of type II, III, and IV patients decreased (Fig. S2a). As time went by, the accumulated ratio of type I patients gradually increased from 25.0% to 70.7%, whereas the accumulated ratios of type II, III, and IV patients gradually decreased (Tables S5–S6 and Fig. S2b).

From our sound sensor monitoring, we proved that the lung and heart injuries in pneumonia patients gradually decreased after discharge, and the lung and heart functions of the patients gradually improved over time. The results of our sensor monitoring were in agreement with clinical observations that pneumonia patients can suffer long-term lung and heart damage, but their condition tends to improve over time^25^.

Based on the above results from sound sensor monitoring, we found four typical characteristics in discharged pneumonia patients (Tables 4 and 5 and Fig. 6), and we found lung injury (14.6%), heart injury (4.9%), and both lung and heart injury (9.8%) is discharged patients (Table 5). Our results were consistent with ECG data (Table 5) and clinical observations based on chest CT^2,3^ and cardiac MRI^8,9^. With our sensor device, we successfully tracked the recovery course of the pneumonia patients. Over time, the lung and heart states of the patients gradually improved after discharge (Figs. 7 and  S2), and our sound sensor observations were in good agreement with the clinically reported tendency^25^.

Compared with conventional large, sophisticated and expensive instruments, our small-sized sensor provides a rapid, simple, highly sensitive approach to detect lung and heart sounds, which greatly helps the evaluation of lung and heart states of pneumonia patients and provides an alternative approach for the diagnosis and prognosis of pneumonia disease. Moreover, our sensor provides a robust approach to capture lung and heart sounds, where patients can reliably obtain the same high-quality signals as trained medical personnel; therefore, our sensor has great potential for clinical use as well as home-use health monitoring, especially in the field of wearable electronics^26,27^.

## Conclusions

In this study, we developed a two-stage amplified PZT sensor for lung and heart sound monitoring in discharged pneumonia patients. Benefiting from the asymmetric gapped cantilever structure and built-in charge amplifier circuit, our accelerometer exhibited an extremely high ratio of sensitivity to noise compared to commercialized accelerometers. In addition, a sensitivity of 9.2 V/g at a frequency less than 1000 Hz was achieved by our sensor, making it suitable for weak lung and heart sound monitoring. We used our ultrasensitive sound sensor to study the lung and heart states of discharged pneumonia patients. According to our sensor monitoring, for the first time, we classified the discharged pneumonia patients into four types: patients with a normal respiratory rate and normal heart rate, patients with shortness of breath but a normal heart rate, patients with a normal respiratory rate but high heart rate, and patients with shortness of breath and a high heart rate, which represented 70.7%, 14.6%, 4.9% and 9.8% of the discharged patients, respectively. With our sound sensor, we successfully tracked the recovery course of pneumonia patients. Over time, the lung and heart function of the patients gradually improved to normal performance after discharge. Compared with conventional medical instruments, our small-sized sensor provides a rapid, simple, and highly sensitive detection of lung and heart sounds, which greatly helps the evaluation of lung and heart states of pneumonia patients. Our sensor device provides a cost-effective alternative approach to the diagnosis and prognosis of pneumonia diseases or other similar diseases and has great potential for clinical use and home-use health monitoring.

## Materials and methods

### Sensor design and working principles

We designed a two-stage amplified PZT sensor with high sensitivity for cardiorespiratory sound monitoring (Fig. 1a, b). First, the sound sensor was based on piezoelectric materials and an asymmetric gapped cantilever structure (Fig. 1c, d), which was composed of a bottom mechanical layer and a top piezoelectric layer separated by a gap (Fig. 1d and Table 1). The top piezoelectric layer was made from ceramic PZT (lead zirconium titanate, Pb(Zr_*x*_Ti_(1-*x*)_)O_3_), a widely used piezoelectric material^28^. Due to the piezoelectric effect of PZT materials, the strain on the piezoelectric beam could be transferred into electric charge^29^. Second, a built-in charge amplifier circuit was designed to further amplify the electric signal produced by the piezoelectric beam (Fig. 1e). A 1 GΩ resistor (*R*_*F*_) and a 47 pF capacitor (*C*_*F*_) were used as the feedback resistor and feedback capacitor, respectively. An ultralow input bias current operational amplifier (LMP7721, Texas Instruments) was used to achieve the best performance of the sensor (Fig. 1e). To minimize the surface current leakage, a guard trace was designed to completely surround the input terminal and other circuitry connecting to the inputs of the operational amplifier. Together, the fabricated prototype sensor had a total weight of 13.5 g with a size of 39 × 23 × 13.5 mm (*l* × *w* × *h*).

### Theoretical simulation of sensor performance

We used theoretical simulation to estimate sensor performance with different structures: one was the accelerometer with asymmetric gapped cantilever structure in the present study (Table 1), and the other was the conventional cantilever structure (Fig. S1). Harmonic analysis of the dynamic model of different structures was performed in COMSOL Multiphysics® (COMSOL Inc.), with the parameters set as described in Table 1. The amplitude-frequency of the accelerometers under different excitation forces were simulated. The amplitude was then converted to the strain, and the strain on the piezoelectric beam was plotted over the frequency with variable excitation forces.

### Measurement of sensitivity of our sensor

We characterized the frequency response of our sensor using a mechanical shaker (ET-126B, Labworks). We used a commercial accelerometer (752A13, Endevco) as the gold standard sensor. The outputs of both sensors were voltage, which was recorded by a 16-bit data acquisition board (NI USB 6210, National Instrument) simultaneously. The voltages were recorded by setting the shaker to different frequencies (0 to 2000 Hz) and different accelerations. The sensitivity of the sensors was calculated over different frequencies.

### Collection of lung and heart signal data

We recorded the lung and heart sounds of healthy volunteers (*n* = 5) and discharged pneumonia patients during the follow-up visit in the hospital (*n* = 41). The pneumonia patients who met the discharge criteria were discharged from the hospital. At different times after discharge (in weeks), the patients visited the hospital for a follow-up examination, and we monitored the lung and heart sounds of patients during their follow-up visit.

During sound monitoring, we chose the device location to the right anterior intercostal space above the level of the third rib for respiratory signal detection and the fifth intercostal space to the left immediately lateral to the sternum for cardiac signal detection (Fig. 1a). We recorded the lung and heart sounds for 60 s for each assay. In this study, data were recorded from 41 pneumonia patients who were recently discharged between 15 June and 2 September 2020. If the same patient was monitored several times on different dates, only the data from the first monitoring session were used for analysis of type classification.

For comparison, we also monitored the lung and heart sounds using a commercial high-end electronic stethoscope (3 M Littman 3200), and the results of both devices were compared.

### Data processing

We transferred the collected data from the sound sensors to a computer through a data acquisition board (NI USB 6210), and we further processed the data by LabVIEW^®^ and MATLAB^®^. We fixed the sampling rates to be 6 kHz. For data treatment, we applied a filter with a bandwidth from 20 to 400 Hz to extract heart sounds and applied a filter with a bandwidth from 60 to 1000 Hz to extract lung sounds.

### Statistical analysis

No statistical methods were used to predetermine sample sizes. The experiments were not randomized, and investigators were not blinded during experiments and outcome assessment. Data are presented as the mean ± standard deviation (SD). Statistical analysis was performed using Student’s *t*-test, and a *p* value less than 0.05 was considered statistically significant.

## Supplementary information

Supplementary Information

Audio S1. Lung sound recorded from a healthy volunteer (15s)

Audio S2. Heart sound recorded from a healthy volunteer (10s)

Audio S3. Lung sound recorded in a patient of Type I (15s)

Audio S4. Heart sound recorded in a patient of Type I (10s)

Audio S5. Lung sound recorded in a patient of Type II (15s)

Audio S6. Heart sound recorded in a patient of Type II (10s)

Audio S7. Lung sound recorded in a patient of Type III (15s)

Audio S8. Heart sound recorded in a patient of Type III (10s)

Audio S9. Lung sound recorded in a patient of Type IV (15s)

Audio S10. Heart sound recorded in a patient of Type IV (10s)
