# Supplementary material for: A two-stage amplified PZT sensor for monitoring lung and heart sounds in discharged pneumonia patients
Source: Microsyst Nanoeng. 2021 Jul 22;7:55. doi: 10.1038/s41378-021-00274-x (PMC8433369; doi:10.1038/s41378-021-00274-x)
Supplement: Supplementary file 1 — Supplementary Information [file 41378_2021_274_MOESM1_ESM.docx]

**Supplementary Information**

**A** **two-stage amplified PZT sensor for lung and heart sound monitoring in discharged patients with pneumonia**

Hongbin Chen ^a,#^, Shuai Yu ^b,#^, Haiyang Liu ^c^, Jie Liu ^c^, Yongguang Xiao ^d^, Dandan Wu ^a^,

Xiaoyu Pan ^e^, Cuihong Zhou ^e^, Yifeng Lei ^c,*^, Sheng Liu ^c,f*^

^a^ Department of Pulmonary and Critical Care Medicine, Renmin Hospital of Wuhan University, Wuhan 430060, China

^b^ School of Mechanical Science and Engineering, Huazhong University of Science and Technology, Wuhan 430074, China

^c^ School of Power and Mechanical Engineering & the Institute of Technological Science, Wuhan University, Wuhan 430072, China

^d^ Department of Thoracic, Renmin Hospital of Wuhan University, Wuhan 430060, China

^e^ Department of Pulmonary and Critical Care Medicine, the Ninth Hospital of Wuhan, Wuhan 430081, China

^f^ School of Microelectronics, Wuhan University, Wuhan 430072, China

^#^ These authors contributed equally to this work

^*^ Corresponding authors. Email address: shengliu@whu.edu.cn (S. Liu), yifenglei@whu.edu.cn (Y. Lei)

**Supplementary Figures**


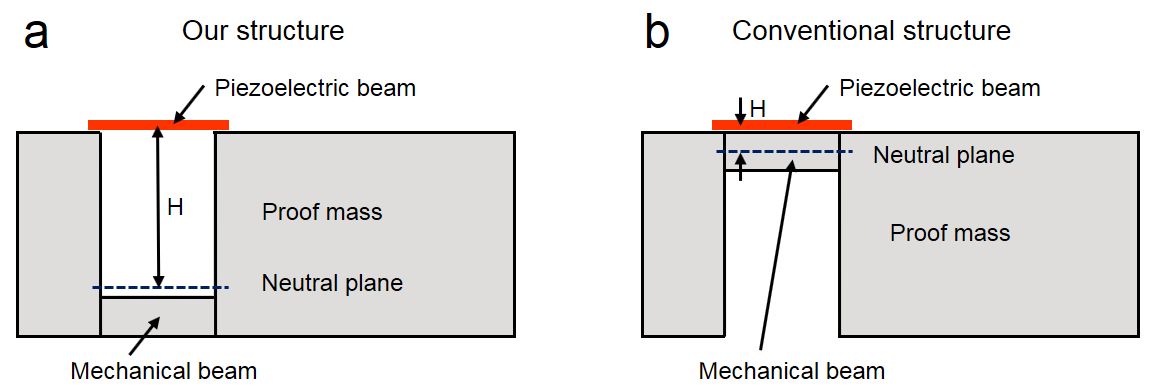


**Fig. S1.** Comparison of different structure designs. (a) Our structure based on asymmetric gapped cantilever. (b) Conventional structure with cantilever structure. H is the distance between the top piezoelectric beam and the neutral plane.


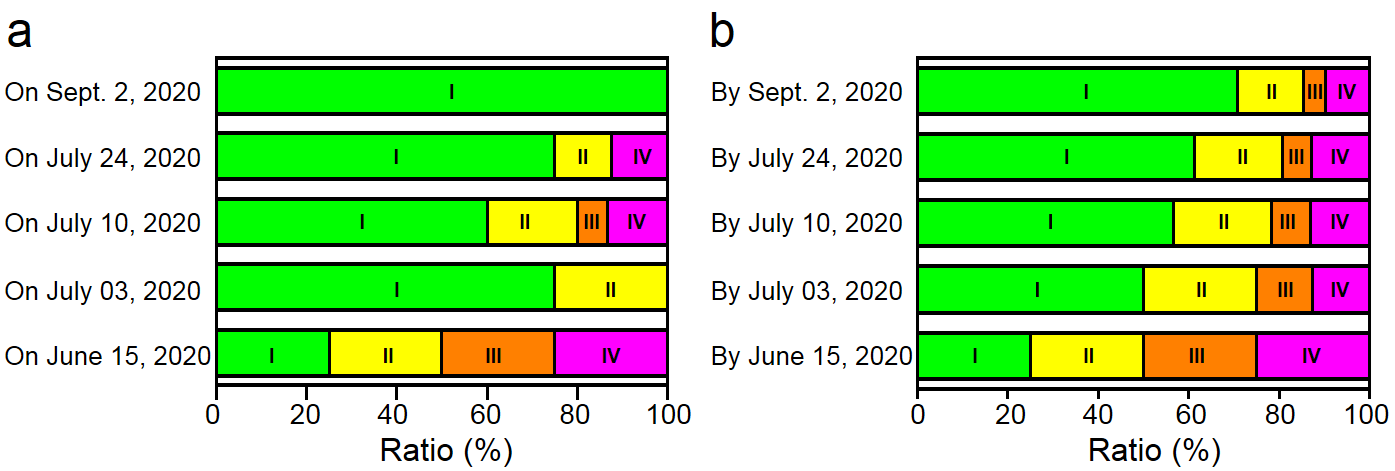


**Fig. S2.** Distribution of four types of lung and heart states in discharged pneumonia patients, with (a) ratio of patients monitored on different dates, (b) accumulated ratio of patients by different dates.

**Supplementary Tables**

**Table S1.** Lung and heart sound recorded in discharged pneumonia patients. ECG data were provided for comparison.

| ID | Respiratory rate by sensor (BPM) | Heart rate by sensor (bpm) | Heart rate by ECG (bpm) | Date | Type classification |
| --- | --- | --- | --- | --- | --- |
| 1# | 15.8 | 69.8 | 70 | June 15, 2020 | I |
| 2# | 12.5 | 88.2 | 87 | June 15, 2020 | III |
| 3# | 24.0 | 68.2 | 69 | June 15, 2020 | II |
| 4# | 30.0 | 113.2 | 105 | June 15, 2020 | IV |
| 5# | 18.2 | 73.2 | 75 | July 3, 2020 | I |
| 6# | 15.4 | 66.7 | 68 | July 3, 2020 | I |
| 7# | 15.8 | 63.2 | 62 | July 3, 2020 | I |
| 8# | 24.0 | 76.9 | 78 | July 3, 2020 | II |
| 9# | 31.6 | 93.8 | 96 | July 10, 2020 | IV |
| 10# | 21.4 | 81.1 | 81 | July 10, 2020 | II |
| 11# | 20.7 | 68.2 | 69 | July 10, 2020 | II |
| 12# | 16.7 | 78.9 | 81 | July 10, 2020 | I |
| 13# | 18.2 | 69.0 | 72 | July 10, 2020 | I |
| 14# | 19.4 | 84.5 | 86 | July 10, 2020 | I |
| 15# | 30.0 | 100.0 | 103 | July 10, 2020 | IV |
| 16# | 13.3 | 89.6 | 92 | July 10, 2020 | III |
| 17# | 14.3 | 68.2 | 68 | July 10, 2020 | I |
| 18# | 19.4 | 69.0 | 70 | July 10, 2020 | I |
| 19# | 16.7 | 69.8 | 71 | July 10, 2020 | I |
| 20# | 16.7 | 84.5 | 84 | July 10, 2020 | I |
| 21# | 14.3 | 82.2 | 82 | July 10, 2020 | I |
| 22# | 17.6 | 73.2 | 71 | July 10, 2020 | I |
| 23# | 23.1 | 83.3 | 80 | July 10, 2020 | II |
| 24# | 17.1 | 73.2 | 74 | July 24, 2020 | I |
| 25# | 18.8 | 76.9 | 77 | July 24, 2020 | I |
| 26# | 24.0 | 77.9 | 80 | July 24, 2020 | II |
| 27# | 15.4 | 80.0 | 82 | July 24, 2020 | I |
| 28# | 28.6 | 88.2 | 87 | July 24, 2020 | IV |
| 29# | 16.7 | 63.2 | 66 | July 24, 2020 | I |
| 30# | 19.4 | 76.9 | 76 | July 24, 2020 | I |
| 31# | 16.7 | 72.3 | 71 | July 24, 2020 | I |
| 32# | 20.0 | 66.7 | 67 | Sept. 2, 2020 | I |
| 33# | 15.8 | 69.0 | 69 | Sept. 2, 2020 | I |
| 34# | 14.6 | 69.8 | 70 | Sept. 2, 2020 | I |
| 35# | 13.0 | 61.9 | 65 | Sept. 2, 2020 | I |
| 36# | 16.2 | 63.2 | 65 | Sept. 2, 2020 | I |
| 37# | 14.3 | 65.9 | 66 | Sept. 2, 2020 | I |
| 38# | 15.8 | 70.6 | 73 | Sept. 2, 2020 | I |
| 39# | 15.0 | 69.0 | 67 | Sept. 2, 2020 | I |
| 40# | 16.2 | 63.2 | 65 | Sept. 2, 2020 | I |
| 41# | 15.4 | 63.8 | 66 | Sept. 2, 2020 | I |

**Table S2.** Tracking of lung and heart sounds of a pneumonia patient (23#) on different dates.

| Date | Respiratory rate by sensor (BPM) | Heart rate by sensor (bpm) | Heart rate by ECG (bpm) |
| --- | --- | --- | --- |
| July 10, 2020 | 23.1 | 83.3 | 80 |
| July 24, 2020 | 18.8 | 76.9 | 78 |
| September 2, 2020 | 16.2 | 74.1 | 75 |

**Table S3.** Number of discharged pneumonia patients on different dates of sensor monitoring.

| Type | On June 15, 2020 | On July 3, 2020 | On July 10, 2020 | On July 24, 2020 | On Sept. 2, 2020 |
| --- | --- | --- | --- | --- | --- |
| I | 1 | 3 | 9 | 6 | 10 |
| II | 1 | 1 | 3 | 1 | 0 |
| III | 1 | 0 | 1 | 0 | 0 |
| IV | 1 | 0 | 2 | 1 | 0 |
| Total | 4 | 4 | 15 | 8 | 10 |

**Table S4.** Ratio of discharged pneumonia patients on different monitoring dates.

| Type | On June 15, 2020 | On July 3, 2020 | On July 10, 2020 | On July 24, 2020 | On Sept. 2, 2020 |
| --- | --- | --- | --- | --- | --- |
| I | 25.0% | 75.0% | 60.0% | 75.0% | 100.0% |
| II | 25.0% | 25.0% | 20.0% | 12.5% | 0.0% |
| III | 25.0% | 0.0% | 6.7% | 0.0% | 0.0% |
| IV | 25.0% | 0.0% | 13.3% | 12.5% | 0.0% |
| Total | 100.0% | 100.0% | 100.0% | 100.0% | 100.0% |

**Table S5.** Accumulated number of discharged pneumonia patients by different dates of monitoring.

| Type | By June 15, 2020 | By July 3, 2020 | By July 10, 2020 | By July 24, 2020 | By Sept. 2, 2020 |
| --- | --- | --- | --- | --- | --- |
| I | 1 | 4 | 13 | 19 | 29 |
| II | 1 | 2 | 5 | 6 | 6 |
| III | 1 | 1 | 2 | 2 | 2 |
| IV | 1 | 1 | 3 | 4 | 4 |
| Total | 4 | 8 | 23 | 31 | 41 |

**Table S6.** Accumulated ratio of discharged pneumonia patients by different monitoring dates.

| Type | By June 15, 2020 | By July 3, 2020 | By July 10, 2020 | By July 24, 2020 | By Sept. 2, 2020 |
| --- | --- | --- | --- | --- | --- |
| I | 25.0% | 50.0% | 56.5% | 61.3% | 70.7% |
| II | 25.0% | 25.0% | 21.7% | 19.4% | 14.6% |
| III | 25.0% | 12.5% | 8.7% | 6.5% | 4.9% |
| IV | 25.0% | 12.5% | 13.0% | 12.9% | 9.8% |
| Total | 100.0% | 100.0% | 100.0% | 100.0% | 100.0% |

**Supplementary Audios**

**Audio S1.** Lung sound recorded from a healthy volunteer (15s).

**Audio S2.** Heart sound recorded from a healthy volunteer (10s).

**Audio S3.** Lung sound recorded in a patient of Type I (15s).

**Audio S4.** Heart sound recorded in a patient of Type I (10s).

**Audio S5.** Lung sound recorded in a patient of Type II (15s).

**Audio S6.** Heart sound recorded in a patient of Type II (10s).

**Audio S7.** Lung sound recorded in a patient of Type III (15s).

**Audio S8.** Heart sound recorded in a patient of Type III (10s).

**Audio S9.** Lung sound recorded in a patient of Type IV (15s).

**Audio S10.** Heart sound recorded in a patient of Type IV (10s).
